# Maternal dyslipidemia and risk for preterm birth

**DOI:** 10.1371/journal.pone.0209579

**Published:** 2018-12-21

**Authors:** Caitlin J. Smith, Rebecca J. Baer, Scott P. Oltman, Patrick J. Breheny, Wei Bao, Jennifer G. Robinson, John M. Dagle, Liang Liang, Sky K. Feuer, Christina D. Chambers, Laura L. Jelliffe-Pawlowski, Kelli K. Ryckman

**Affiliations:** 1 Department of Epidemiology, University of Iowa, Iowa City, Iowa, United States of America; 2 Department of Pediatrics, University of California San Diego, La Jolla, California, United States of America; 3 California Preterm Birth Initiative, University of California San Francisco, San Francisco, California, United States of America; 4 Department of Epidemiology and Biostatistics, University of California San Francisco, San Francisco, California, United States of America; 5 Department of Biostatistics, University of Iowa, Iowa City, Iowa, United States of America; 6 Department of Pediatrics, University of Iowa, Iowa City, Iowa, United States of America; 7 Department of Genetics, Stanford University, Stanford, California, United States of America; 8 Department of Obstetrics, Gynecology & Reproductive Sciences, University of California San Francisco, San Francisco, California, United States of America; Mount Sinai Health System, University of Toronto, CANADA

## Abstract

Maternal lipid profiles during pregnancy are associated with risk for preterm birth. This study investigates the association between maternal dyslipidemia and subsequent preterm birth among pregnant women in the state of California. Births were identified from California birth certificate and hospital discharge records from 2007–2012 (N = 2,865,987). Preterm birth was defined as <37 weeks completed gestation and dyslipidemia was defined by diagnostic codes. Subtypes of preterm birth were classified as preterm premature rupture of membranes (PPROM), spontaneous labor, and medically indicated, according to birth certificate data and diagnostic codes. The association between dyslipidemia and preterm birth was tested with logistic regression. Models were adjusted for maternal age at delivery, race/ethnicity, hypertension, pre-pregnancy body mass index, insurance type, and education. Maternal dyslipidemia was significantly associated with increased odds of preterm birth (adjusted OR: 1.49, 95%CI: 1.39, 1.59). This finding was consistent across all subtypes of preterm birth, including PPROM (adjusted OR: 1.54, 95%CI: 1.34, 1.76), spontaneous (adjusted OR: 1.51, 95%CI: 1.39, 1.65), and medically indicated (adjusted OR: 1.454, 95%CI: 1.282, 1.649). This study suggests that maternal dyslipidemia is associated with increased risk for all types of preterm birth.

## Introduction

Preterm birth is defined as delivery prior to 37 weeks of completed gestation. The World Health Organization estimates that preterm birth affects 11% of pregnancies worldwide, representing nearly 15 million births in 2010 [1]. It is the second leading cause of death in children under age 5 [1]. Despite decades of research into the causes of preterm birth, the biological causes of preterm birth remain largely unknown [2].

Normal pregnancy is accompanied by metabolic changes, particularly in carbohydrate and lipid metabolism. The benefit of these changes is presumably to increase circulating glucose and triglycerides to nourish the growing fetus. Changes in carbohydrate metabolism are bimodal, in which fasting plasma glucose is decreased in early pregnancy, and impaired glucose tolerance occurs in late pregnancy [3]. Circulating lipids, including high density lipoprotein (HDL), low density lipoprotein (LDL), total cholesterol, and triglycerides, increase throughout pregnancy, with the greatest increase observed for triglycerides [3]. Although much research has been devoted to glucose metabolism during pregnancy due to the risk of gestational diabetes mellitus [4], increasing interest in lipid levels during pregnancy has revealed associations between maternal lipid levels and adverse pregnancy outcomes, including preterm birth.

Many studies have investigated associations between maternal lipid levels during pregnancy and risk for preterm birth, although the lipid components and magnitude of associations have been inconsistent across studies [5–17]. One previous study investigated the association between dyslipidemia, as defined by lipid levels in prenatal screening, and found increased risks for preterm birth with mid-trimester hyperlipidemia in combination with elevated levels of tumor necrosis alpha [8]. The present study investigates the association between a clinical diagnosis of maternal dyslipidemia and subsequent preterm birth among pregnant women in the state of California.

## Materials and methods

### Study population

Births were identified from California birth certificate and hospital discharge records from 2007–2012 (N = 2,962,434) as collected by the California Office of Statewide Health Planning and Development. Records were linked approximately 9–12 months prior to delivery through 9–12 months post-delivery [18]. Inclusion criteria included singleton pregnancy, availability of linked records, gestational age between 20–44 weeks and absence of severe hypertensive diseases including hypertensive heart disease, hypertensive chronic kidney disease and secondary hypertension. We excluded these women, as those forms of hypertension are not the primary focus of our study and could confound the association with preterm birth. Dyslipidemia was defined by the International Classification of Diseases and Related Health Problems (ICD-9) codes 272.0–272.4. Specifically, these codes include pure hypercholesterolemia (ICD-9 272.0), pure hyperglyceridemia (ICD-9 272.1), mixed hyperlipidemia (ICD-9 272.2), hyperchylomicronemia (ICD-9 272.3), and other unspecified hyperlipidemia (ICD-9 272.4). We restricted our dyslipidemia definitions to only those that occurred on a hospital admission at or prior to the delivery date. Methods and protocols were approved by the Committee for the Protection of Human Subjects within the Health and Human Services Agency of the State of California. All data was de-identified and determined not to qualify as human subjects research by the University of Iowa Institutional Review Board.

### Core outcomes and statistical analysis

Preterm birth was defined as gestational age at delivery <37 weeks and term birth was defined as gestational age at delivery ≥37 weeks, according to best obstetric estimate. Births were further categorized into early preterm birth (<32 weeks), late preterm birth (32–36 67 weeks) and term birth (≥37 weeks). Subtypes of preterm birth were classified as preterm premature rupture of membranes (PPROM), spontaneous, and medically indicated, according to birth certificate data or hospital discharge records as previously described [19]. Specifically, preterm births with indication of premature rupture of membranes were classified as PPROM and births with indication of preterm labor or tocolytic medication AND absence of PPROM were classified as spontaneous. Births with absence of premature rupture of membranes, premature labor and tocolytic medication AND a code for ‘medical induction’ or ‘artificial rupture of membranes’ or cesarean delivery without such codes were classified as medically indicated.

All analyses were performed using Statistical Analysis Software (SAS) version 9.4 (SAS Institute, Cary, North Carolina). The associations between dyslipidemia and preterm birth were tested using logistic regression (PROC LOGISTIC). Dyslipidemia was modeled as a composite variable and as individual diagnostic codes. The associations were tested without adjustment and with adjustment for maternal age at delivery, hypertension (which included pre-existing essential hypertension, gestational hypertension, pre-eclampsia or eclampsia), race/ethnicity, BMI, insurance type, and education. Maternal age at delivery was analyzed as a linear variable. Hypertension was coded as a binary variable and the absence of hypertension was used as the referent group. Race/ethnicity was categorized as Black, Asian, Caucasian or Hispanic, and Caucasian was used as the referent group. BMI was categorized according to standard cut-points (underweight [<18.5], normal [18.5–24.9], overweight [25–29.9], or obese [≥30]), and ‘normal’ was used as the referent group [20]. Insurance type was categorized as Medi-Cal, private, self-pay, or other, and ‘private’ was used as the referent group. Medi-Cal is California’s Medicaid program, which provides health insurance and health care services for low-income individuals. Education was categorized as <12 years, exactly 12 years (completion of high school diploma), or >12 years, which was used as the referent group.

We considered maternal age at delivery as a potential confounder, wherein we hypothesized that advanced maternal age would be associated with increased likelihood of diagnosis of dyslipidemia and an increased likelihood of delivering preterm [21–24]. We also considered BMI as a potential confounder, in which overweight and obesity would be associated with increased likelihood of diagnosis of dyslipidemia and increased likelihood of delivering preterm [23–26]. Other potential confounders including race/ethnicity, maternal age at delivery, hypertension (includes both pre-pregnancy and pregnancy diagnoses), pre-pregnancy body mass index (BMI), insurance type, and education [21–26]. These variables were available from birth certificate records. Hypertension diagnoses were also confirmed by hospital discharge records.

Several supplemental analyses were performed. These included: 1) stratification of analyses by BMI category to determine if BMI modifies the relationship between dyslipidemia and preterm birth; 2) consolidation of ICD-9 codes into cholesterol dyslipidemia and triglyceride dyslipidemia to determine if the type of dyslipidemia (cholesterol versus triglyceride) affects the results; and 3) examination of the individual impact of confounders, including race/ethnicity, hypertension, BMI, insurance type, maternal education and maternal age, on the association between dyslipidemia and preterm birth. All statistical analyses were performed with statistical power >99.9% to detect an odds ratio of 1.5.

## Results

Demographic characteristics of the study population are presented in Table 1. The analysis included 9,162 women with dyslipidemia and 2,953,272 women without dyslipidemia. Women with dyslipidemia differed from women without dyslipidemia by race/ethnicity, BMI, hypertension (pre-existing or onset during pregnancy), insurance status, education, and maternal age at delivery (all at p<0.0001). Specifically, the group of women who had dyslipidemia included more Black women, were less likely to have a normal BMI, were more likely to have hypertension, were less likely to be on Medi-Cal insurance, were more likely to have completed more than 12 years of education, and were slightly older than the group of women without dyslipidemia.

**Table 1:** Demographic characteristics of study population.

	Dyslipidemia (N = 9,162)	No Dyslipidemia (N = 2,953,272)
Maternal Age at Delivery*	32.4 ± 5.97	28.3 ± 6.29
Race		
*Black*	646 (7.8%)	157,917 (5.8%)
*Asian*	1,305 (15.7%)	365,274 (13.4%)
*Caucasian*	2,293 (27.5%)	770,805 (28.2%)
*Hispanic*	4,086 (49.0%)	1,963,803 (52.7%)
*Missing (N = 218*,*664)*		
BMI		
*Underweight*	190 (2.2%)	144,146 (5.2%)
*Normal*	2,278 (26.7%)	1,349,503 (49.0%)
*Overweight*	2,308 (27.1%)	701,674 (25.5%)
*Obese*	3,754 (44.0%)	558,825 (20.3%)
*Missing (N = 199*,*124)*		
Insurance		
*MediCal*	2,473 (27.0%)	1,427,199 (48.4%)
*Private*	6413 (70.1%)	1,366,516 (46.4%)
*Self-Pay*	58 (0.6%)	59,778 (2.0%)
*Other*	210 (2.3%)	95,014 (3.2%)
*Missing (N = 4*,*765)*		
Education		
*<12 years*	1,350 (15.3%)	707,119 (24.9%)
*12 completed years*	2,208 (25.0%)	755,196 (26.6%)
*>12 years*	5,292 (59.8%)	1,381,901 (48.6%)
*Missing (N = 109*,*056)*		
Hypertension		
*Yes*	2,447 (26.7%)	209,004 (7.1%)
Preterm		
*Yes*	1,369 (14.9%)	209,717 (7.1%)

*Data are presented as mean ± standard deviation. All other data are presented as N (%).

Results of the traditional logistic regression analyses are presented in Table 2. Three different outcomes are presented: preterm versus term, early and late preterm versus term, and PPROM, spontaneous, and medically indicated versus term. Dyslipidemia was significantly associated with preterm birth, both before and after adjusting for race/ethnicity, maternal age at delivery, hypertension, BMI, insurance type, and education.

**Table 2:** Association between dyslipidemia and preterm birth.

	Total population (N = 2,962,434)	
	Unadjusted OR (95% CI)	Adjusted* OR (95% CI)
Outcome 1^a^	2.30 (2.17, 2.44)	1.49 (1.39, 1.59)
Outcome 2^b^		
*<32 weeks vs*. *Term*	2.97 (2.61, 3.37)	1.63 (1.41, 1.89)
*32–36 weeks vs*. *Term*	2.19 (2.06, 2.33)	1.46 (1.36, 1.57)
Outcome 3		
*PPROM vs*. *term*	1.92 (1.69, 2.17)	1.54 (1.34, 1.76)
*Spon*. *vs*. *term*	2.43 (2.26, 2.62)	1.51 (1.39, 1.65)
*Indicated vs*. *term*	2.85 (2.55, 3.17)	1.45 (1.28, 1.65)

*Adjusted for race, maternal age at delivery, hypertension, body mass index, insurance type, and education

^a^Preterm birth (<37 weeks) vs. term birth (≥37 weeks)

^b^Early and late preterm birth

Results of the traditional logistic regression analyses, stratified by type of dyslipidemia, are presented in Table 3. Hyperchylomicronemia (ICD-9 272.3) was not analyzed due to low sample size (N<10). Each type of dyslipidemia was significantly associated with preterm birth, both before and after adjusting for race/ethnicity, maternal age at delivery, hypertension, BMI, insurance type, and education.

**Table 3:** Association between types of dyslipidemia and preterm birth.

	Pure hypercholesterolemia (N = 2,599)		Pure hyperglyceridemia (N = 6,81)		Mixed hyperlipidemia (N = 379)		Other unspecified hyperlipidemia (N = 6,088)		Maternal lipid disorder before delivery (N = 6,816)	
	UnaOR (95% CI)	AOR* (95% CI)	UnaOR (95% CI)	AOR* (95% CI)	UnaOR (95% CI)	AOR* (95% CI)	UnaOR (95% CI)	AOR* (95% CI)	UnaOR (95% CI)	AOR* (95% CI)
Outcome 1^a^	2.16 (1.93, 2.41)	1.30 (1.14, 1.47)	2.54 (2.07, 3.12)	1.64 (1.29, 2.09)	2.41 (1.82, 3.18)	1.77 (1.29, 2.43)	2.39 (2.23, 2.57)	1.53 (1.41, 1.66)	2.32 (2.17, 2.48)	1.63 (1.50, 1.76)
Outcome 2^b^										
*<32 weeks vs*. *Term*	2.92 (2.30, 3.70)	1.40 (1.07, 1.83)	3.43 (2.22, 5.31)	2.07 (1.26, 3.39)	2.94 (1.57, 5.50)	2.03 (1.03, 3.99)	3.07 (2.63, 3.58)	1.67 (1.40, 2.00)	2.89 (2.49, 3.36)	1.79 (1.51, 2.13)
*32–36 weeks vs*. *Term*	2.04 (1.81, 2.30)	1.28 (1.11, 1.46)	2.39 (1.92, 2.99)	1.56 (1.20, 2.03)	2.32 (1.72, 3.14)	1.72 (1.22, 2.42)	2.28 (2.12, 2.46)	1.50 (1.38, 1.64)	2.23 (2.07, 2.39)	1.59 (1.47, 1.73)
Outcome 3										
*PPROM vs*. *term*	1.64 (1.28, 2.11)	1.41 (1.08, 1.83)	2.05 (1.31, 3.21)	1.86 (1.16, 2.99)	1.81 (0.97, 3.40)	1.43 (0.71, 2.88)	2.06 (1.77, 2.39)	1.57 (1.33, 1.86)	1.99 (1.73, 2.29)	1.61 (1.38, 1.89)
*Spon*. *vs*. *term*	2.43 (2.11, 2.79)	1.38 (1.17, 1.61)	3.23 (2.53, 4.13)	1.76 (1.30, 2.40)	2.59 (1.81, 3.71)	1.99 (1.33, 2.97)	2.42 (2.21, 2.65)	1.50 (1.34, 1.67)	2.45 (2.25, 2.68)	1.70 (1.54, 1.88)
*Indicated vs*. *term*	2.46 (1.98, 3.05)	1.13 (0.89, 1.44)	2.50 (1.63, 3.83)	1.56 (0.99, 2.46)	3.05 (1.82, 5.10)	1.62 (0.88, 2.98)	3.23 (2.85, 3.66)	1.65 (1.43, 1.91)	2.84 (2.50, 3.22)	1.53 (1.32, 1.77)

*AOR: Adjusted odds ratio including race, maternal age at delivery, hypertension, body mass index, insurance type, and education

^a^Preterm birth (<37 weeks) vs. term birth (≥37 weeks)

^b^Early and late preterm birth

Results of the traditional logistic regression analyses, stratified by BMI category, are presented in S1 Table. Within each BMI category, dyslipidemia was significantly associated with preterm birth. After adjusting for maternal age at delivery, hypertension, race/ethnicity, insurance type, and education, dyslipidemia was significantly associated with preterm birth among normal weight, overweight, and obese women, but not among underweight women. Obesity itself was associated with a 1.6-fold increase in risk for medically indicated preterm birth compared to normal BMI (OR: 1.61; 95%CI: 1.57, 1.65).

Results of the consolidation of ICD-9 codes into cholesterol dyslipidemia and triglyceride dyslipidemia are presented in S2 Table. Cholesterol dyslipidemia, which included pure hypercholesterolemia and mixed dyslipidemia, was significantly associated with preterm birth before and after adjustment. Triglyceride dyslipidemia, which included pure hyperglyceridemia and hyperchylomicronemia, was significantly associated with preterm birth before and after adjustment.

To investigate the individual impact of confounders, including race/ethnicity, hypertension, BMI, insurance type, maternal education and maternal age, on the association between dyslipidemia and preterm birth, each confounder was individually added to the logistic regression models (S3 Table). Adjusting for hypertension alone showed the greatest attenuation of the association between dyslipidemia and preterm birth of all the individual confounders (OR: 1.53; 95%CI: 1.45, 1.63). Adjusting for other confounders did not affect the odds ratios compared to the unadjusted models.

## Discussion

In this retrospective cohort of 2.9 million pregnant women in California, maternal diagnosis of dyslipidemia was significantly associated with increased risk for preterm birth. To the best of our knowledge, this study represents the largest investigation of the association between clinical dyslipidemia and risk for preterm birth done to date and it is the only study that we know of to utilize hospital diagnostic codes to define dyslipidemia, which include both familial and non-familial forms of dyslipidemia. The size and diversity of the study population allowed for the investigation of the association between dyslipidemia and preterm birth, stratified by subtypes of dyslipidemia.

Several previous studies have investigated associations between maternal lipid levels during pregnancy and risk for preterm birth [5–17]. These studies varied in the lipid components they measured, the gestational age at which they were measured, and fasting status, which may explain their discordant findings. For example, of the seven studies that measured all four lipid components [5–11], four studies failed to identify an association between individual lipid components and risk for preterm birth. Of the three studies that measured only total cholesterol (TC) [15–17] one identified a positive association between elevated TC and preterm birth and two identified associations between both low and high TC and preterm birth. A recent meta-analysis identified significant pooled associations between elevated TC, elevated TG and low HDL and preterm birth [27]. All previous studies have used lipid levels as a continuous exposure, although some categorized lipid levels by percentiles. However, this does not mean that these studies sampled women who would have met criteria for dyslipidemia. Thus, our study is unique in its use of a clinically significant exposure.

Of particular interest in the present study is the consistency of the magnitude of association across all subtypes of preterm birth, after adjusting for potential confounders. These adjusted odds ratios ranged from 1.4–1.6 (Table 2), providing strong and consistent evidence that women with maternal dyslipidemia are approximately one-and-a-half times more likely to deliver preterm than comparable women without dyslipidemia regardless of preterm birth subtype.

Dyslipidemia is often comorbid with obesity [26], and obesity has long been known to increase the risk for pregnancy complications such as gestational diabetes mellitus and preeclampsia [28]. Dyslipidemia severe enough to warrant a clinical diagnosis may be a marker for more severely disturbed cardiometabolic milieu. Chronic dyslipidemia is accompanied by inflammation, a hallmark of obesity, and acute inflammation triggers altered lipid metabolism [29]. Interestingly, stratification by BMI category did not significantly alter the associations between dyslipidemia and preterm birth among overweight or obese women (S1 Table). However, hypertension had the most affect in attenuating the association. Hypertension is known to co-occur with dyslipidemia and increases the risk for preterm birth. This suggests that dyslipidemia is associated with increased risk for preterm birth independent of obesity and some of that risk is explained by the co-occurrence of hypertension.

A limitation of this study is the lack of information regarding dyslipidemia diagnostic practices. Heterogeneity exists among practitioners in terms of the degree of follow-up testing of lipid levels. Thus, some women may have received a diagnosis after a single abnormal lipid panel, with no repeat testing, while other women may have received a diagnosis following multiple abnormal panels. Some women with dyslipidemia may not have a diagnosis because they have never had their lipid levels tested or they were treated before pregnancy and entered pregnancy with normal lipids. This type of non-differential misclassification would bias the results toward the null. There are currently, no-evidence based standards for how to treat women with dyslipidemia during pregnancy. The most common recommendations include lifestyle changes, glycemic control and close follow-up of the pregnancy [23]. It is unlikely that women were treated with cholesterol-lowering drugs such as statins or niacin, since these drugs are contraindicated during pregnancy [30].

It should also be noted that an important limitation of the study is the lack of lipid level information. Such data would have allowed for discrimination between familial, monogenic dyslipidemias, which are characterized by markedly abnormal lipid levels, and non-familial, polygenic dyslipidemias, which typically manifest as less drastic changes in lipid levels. However, a Norwegian study of 895 women with familial hypercholesterolemia (FH) found no association between FH and risk for adverse pregnancy outcomes, including preterm birth [31]. Thus, we can infer that the association between pure hypercholesterolemia and preterm birth is driven by the non-familial form, which may be exacerbated by the co-occurrence of hypertension. Additionally, assuming a prevalence of 1 in 250 for heterozygous FH [32], only ten women with pure hypercholesterolemia would be expected to have FH in our study, which would likely not influence the results. Further, the detection of small differences in lipids between women who deliver term and preterm is unlikely to be clinically meaningful. In contrast, dyslipidemia is a clinically-validated medical condition that could be readily identified as a risk factor for preterm birth. We were also limited to the accuracy of the data on both the hospital discharge record and the birth certificate record, which could have introduced some bias in the estimates of our confounders. There were large amounts of missing data for maternal age, obesity and education; however, when these variables were considered individually, there was little difference between the unadjusted and adjusted models. Therefore, missing data is unlikely to affect our conclusions.

In conclusion, dyslipidemia, as both an aggregate exposure and individual subtypes, was significantly associated with a 1.5-fold increased risk for preterm birth after adjusting for potential confounders. These findings suggest that dyslipidemia may be a potential factor in the etiology of preterm birth, and may serve as a marker of increased risk for preterm birth. The identification of dyslipidemia as a risk factor for preterm birth is impactful for several reasons: 1) There are few known causal risk factors for preterm birth, as the causes of parturition and preterm birth remain largely unknown, 2) dyslipidemia may be modified by lifestyle changes and medication [30] and 3) severe dyslipidemia receiving a clinical diagnosis may be easy to incorporate into clinical decision-making in the era of electronic medical records. Findings from this study support lipid screening among women of reproductive age and additional studies are needed to determine if diagnosing and treating dyslipidemia early in pregnancy reduces the risk for preterm birth.

## Supporting information

S1 Table Association between dyslipidemia and preterm birth, stratified by BMI category. (DOCX)

S2 Table Analysis of consolidation of ICD-9 dyslipidemia codes. (DOCX)

S3 Table Analysis of the individual impact of confounders. (DOCX)
