# Supplementary material for: Maternal dyslipidemia and risk for preterm birth
Source: PLoS One. 2018 Dec 21;13(12):e0209579. doi: 10.1371/journal.pone.0209579 (PMC6303099; doi:10.1371/journal.pone.0209579)
Supplement: S1 Table — (DOCX) [file pone.0209579.s001.docx]

|  | **Underweight (N=144,336)** | | **Normal (N=1,351,781)** | | **Overweight (N=703,982)** | | **Obese (N=562,579)** | |
| --- | --- | --- | --- | --- | --- | --- | --- | --- |
|  | **Unadjusted OR (95% CI)** | **Adjusted***  **OR (95% CI)** | **Unadjusted OR (95% CI)** | **Adjusted***  **OR (95% CI)** | **Unadjusted OR (95% CI)** | **Adjusted***  **OR (95% CI)** | **Unadjusted OR (95% CI)** | **Adjusted***  **OR (95% CI)** |
| **Outcome 1^a^** | **2.30 (1.55, 3.42)** | **1.59 (1.02, 2.48)** | **2.17 (1.91, 2.46)** | **1.50 (1.30, 1.73)** | **2.43 (2.16, 2.73)** | **1.55 (1.36, 1.76)** | **2.27 (2.08, 2.48)** | **1.40 (1.27, 1.55)** |
| **Outcome 2^b^** |  |  |  |  |  |  |  |  |
| ***<32 weeks vs. Term*** | 2.249 (0.833, 6.073) | 1.412 (0.505, 3.952) | **3.380 (2.602, 4.391)** | **2.045 (1.527, 2.737)** | **2.925 (2.260, 3.786)** | **1.560 (1.166, 2.087)** | **2.455 (2.020, 2.983)** | **1.415 (1.144, 1.751)** |
| ***32-36 weeks vs. Term*** | **2.075 (1.361, 3.164)** | 1.474 (0.918, 2.366) | **1.911 (1.669, 2.190)** | **1.411 (1.214, 1.639)** | **2.204 (1.943, 2.501)** | **1.481 (1.287, 1.703)** | **2.181 (1.983, 2.398)** | **1.382 (1.244, 1.537)** |
| **Outcome 3** |  |  |  |  |  |  |  |  |
| ***PPROM vs. normal*** | 1.42 (0.53, 3.84) | 1.01 (0.32, 3.19) | **1.73 (1.32, 2.25)** | **1.41 (1.06, 1.89)** | **2.32 (1.83, 2.90)** | **1.89 (1.48, 2.41)** | **1.76 (1.45, 2.14)** | **1.36 (1.01, 1.68)** |
| ***Spon. vs. normal*** | **2.84 (1.81, 4.48)** | **2.02 (1.21, 3.36)** | **2.37 (2.03, 2.77)** | **1.67 (1.40, 1.98)** | **2.38 (2.04, 2.78)** | **1.42 (1.19, 1.69)** | **2.33 (2.08, 2.61)** | **1.38 (1.21, 1.57)** |
| ***Indicated vs. normal*** | 1.69 (0.62, 4.55) | 1.04 (0.38, 2.88) | **2.17 (1.66, 2.83)** | 1.17 (0.86, 1.59) | **2.67 (2.14, 3.33)** | **1.53 (1.20, 1.95)** | **2.63 (2.25, 3.08)** | **1.48 (1.25, 1.76)** |

**Supplemental Table 1**. Association between dyslipidemia and preterm birth, stratified by BMI category.

*Adjusted for maternal age at delivery, race, hypertension, insurance type, and education ^a^Preterm birth defined by ICD-9 codes
^b^Preterm birth defined by gestational age
