# Supplementary material for: Maternal dyslipidemia and risk for preterm birth
Source: PLoS One. 2018 Dec 21;13(12):e0209579. doi: 10.1371/journal.pone.0209579 (PMC6303099; doi:10.1371/journal.pone.0209579)
Supplement: S2 Table — (DOCX) [file pone.0209579.s002.docx]

| **Dyslipidemia Type** | **Unadjusted OR (95% CI)** | **Adjusted OR (95% CI)** |
| --- | --- | --- |
| Cholesterol (272.0, 272.2) N=2,965 | 2.178 (1.97, 2.41) | 1.343 (1.20, 1.51) |
| Triglyceride (272.1, 272.3) N=683 | 2.560 (2.09, 3.14) | 1.639 (1.29, 2.09) |

**Supplemental Table 2**. Analysis of consolidation of ICD-9 dyslipidemia codes.
