# Supplementary material for: Maternal dyslipidemia and risk for preterm birth
Source: PLoS One. 2018 Dec 21;13(12):e0209579. doi: 10.1371/journal.pone.0209579 (PMC6303099; doi:10.1371/journal.pone.0209579)
Supplement: S3 Table — (DOCX) [file pone.0209579.s003.docx]

| **Outcome** | **BMI** | **Hypertension** | **Race** | **Insurance** | **Education** | **Maternal Age** |
| --- | --- | --- | --- | --- | --- | --- |
| **Outcome 1^a^** | 2.21 (2.08, 2.35) | 1.54 (1.45, 1.63) | 2.25 (2.12, 2.39) | 2.37 (2.24, 2.51) | 2.34 (2.20, 2.48) | 2.22 (2.10, 2.35) |
| **Outcome 2^b^** |  |  |  |  |  |  |
| ***<32 weeks vs. Term*** | 2.76 (2.42, 3.15) | 1.61 (1.41, 1.84) | 2.94 (2.57, 3.37) | 3.15 (2.78, 3.58) | 3.03 (2.66, 3.46) | 2.90 (2.55, 3.29) |
| ***32-36 weeks vs. Term*** | 2.12 (1.98, 2.26) | 1.52 (1.42, 1.62) | 2.14 (2.00, 2.28) | 2.25 (2.11, 2.39) | 2.23 (2.09, 2.37) | 2.11 (1.98, 2.25) |
| **Outcome 3** |  |  |  |  |  |  |
| ***PPROM vs. normal*** | 1.88 (1.66, 2.14) | 1.67 (1.47, 1.89) | 1.88 (1.65, 2.14) | 1.87 (1.65, 2.12) | 1.89 (1.67, 2.15) | 1.77 (1.56, 1.56) |
| ***Spon. vs. normal*** | 2.36 (2.18, 2.55) | 1.53 (1.41, 1.65) | 2.36 (2.18, 2.56) | 2.55 (2.36, 2.75) | 2.49 (2.30, 2.69) | 2.40 (2.23, 2.59) |
| ***Indicated vs. normal*** | 2.52 (2.25, 2.83) | 1.65 (1.48, 1.85) | 2.80 (2.50, 3.19) | 2.92 (2.62, 3.26) | 2.85 (2.55, 3.19) | 2.54 (2.28, 2.84) |

**Supplemental Table 3**. Analysis of the individual impact of confounders.

^a^Preterm birth defined by gestational age
^b^Early and late preterm birth
